# Selection of Reliable Reference Genes in *Caenorhabditis elegans* for Analysis of Nanotoxicity

**DOI:** 10.1371/journal.pone.0031849

**Published:** 2012-03-15

**Authors:** Yanqiong Zhang, Dongliang Chen, Michael A. Smith, Baohong Zhang, Xiaoping Pan

**Affiliations:** Department of Biology, East Carolina University, Greenville, North Carolina, United States of America; University of Kansas, United States of America

## Abstract

Despite rapid development and application of a wide range of manufactured metal oxide nanoparticles (NPs), the understanding of potential risks of using NPs is less completed, especially at the molecular level. The nematode *Caenorhabditis elegans* (C.*elegans*) has been emerging as an environmental model to study the molecular mechanism of environmental contaminations, using standard genetic tools such as the real-time quantitative PCR (RT-qPCR). The most important factor that may affect the accuracy of RT-qPCR is to choose appropriate genes for normalization. In this study, we selected 13 reference gene candidates (act-1, cdc-42, pmp-3, eif-3.C, actin, act-2, csq-1, Y45F10D.4, tba-1, mdh-1, ama-1, F35G12.2, and rbd-1) to test their expression stability under different doses of nano-copper oxide (CuO 0, 1, 10, and 50 µg/mL) using RT-qPCR. Four algorithms, geNorm, NormFinder, BestKeeper, and the comparative ΔCt method, were employed to evaluate these 13 candidates expressions. As a result, tba-1, Y45F10D.4 and pmp-3 were the most reliable, which may be used as reference genes in future study of nanoparticle-induced genetic response using *C.elegans*.

## Introduction

Nanoparticles (NPs), defined as particles with the size of <100 nanometers, display unique physicochemical properties such as size-related high surface reactivity and electrical charges. As a result, manufactured NPs are desirable and widely used for applications in optical, commercial, biomedical, and environmental fields nowadays. Increasing human and wildlife exposure to NPs is expected because of the anticipated increase in utilization of these new materials. However, the biological effects of NPs have been largely unclear, especially at molecular level. Model nanotoxicity studies on TC_60_, carbon nanotubes, and metal oxide NPs reveal oxidative stress-related effects including cytotoxicity, metabolism alterations, and genotoxicity [1]–[4].

Our previous study has used the Ames reverse mutation assay to test the cytotoxicity and mutagencity of several metal oxide nanoparticles including TiO_2_, ZnO, and CuO, etc. [5]. We observed dose–dependent cytotoxic effects of CuO to the *E.coli* strain pKM 101, which is especially sensitive to oxidative stress. Several others' studies also showed the CuO NPs is among the most cytotoxic metal oxide NPs [6]–[8]. Future studies may focus on the molecular effects of metal oxide nanoparticles.

The nematode *Caenorhabditis elegans* (*C. elegans*), a simple and well-defined genetic model, has gained increasing popularity among scientists to study the molecular mechanism of emerging materials. Nematodes are the most abundant soil-dwelling invertebrates that occupied a key position in terrestrial ecosystem by influencing energy transfer and nutrient cycling. *C. elegans*, a free-living nematode that feeds on soil microorganisms, is a simple multicellular eukaryote with its genome first completely sequenced and its cell lineage well-described. *C. elegans* has a short life span, is easy to culture in the laboratory, either in aqueous or in soil matrices. Furthermore, the genome of *C. elegans* showed a high level of conservation with human's genome. All of these advantages make *C. elegans* an ideal test organism for human health and ecological risk assessment by using multiple toxic endpoints, such as behavior, growth, reproduction, and gene expression. Researchers have used *C.elegans* in ecological risk assessment for environmental chemicals including metals [9], [10], pesticides [11], persistent organic pollutants [12], and nanomaterials [13], suggesting *C.elegans* is a sensitive bio-indicator of ecological health effects. The merits of *C. elegans* as both an ecological and a genetic model made it an attractive experimental organism to scientists.

When studying the gene expression, the dominant quantitative method is real-time quantitative PCR (RT-qPCR), which is a highly sensitive technique for precisely measuring the gene expression of various biological specimens. However, this level of sensitivity requires a careful normalization of the expression data between samples. Different normalization strategies are available, but the most common and appropriate one is to apply reference genes as internal controls [14], [15]. However, expression variation in reference genes between different samples and/or under different treatment conditions would significantly affect the expression alteration analysis of genes of interest [16]. Therefore the use of appropriate reference genes for normalization is of fundamental importance in RT-qPCR experiments. However, there is no universal reference gene that could be stably expressed under all experimental conditions. Thus, identification of the reliable reference genes is a prerequisite in RT-qPCR experiments especially when testing the effects of new groups of chemicals. So far, there is no report regarding the identification of the stably expressed reference genes for nanoparticle toxicity studies in *C. elegans*. The aim of this study was to identify and validate a set of reference genes for gene expression analysis in *C. elegans* exposed to NPs, using CuO NPs as the tested compound.

## Materials and Methods

### Nematodes cultivation

*C. elegans* transgenic strain KC136 (hsp16-2–gfp), an oxidative stress sensitive strain, was used. This strain was kindly provided by Dr. King L. Chow from Hong Kong University of Science and Technology. Worms were maintained on NGM (Nematode-Growth-Medium) agar plates seeded with *E. coli* strain OP50 as food, in a 20 degree incubator according to the standard method previously described by Brenner [17]. L4 stage larvae from an age-synchronized culture were used in all the experiments. To obtain age-synchronized cultures, eggs from 3 days of the mature adults plates were isolated via bleaching, followed by rinse with M9 buffer (3 g KH_2_PO_4_, 6 g Na_2_HPO_4_, 5 g NaCl, 1 ml 1 M MgSO_4_, H_2_O to 1 litre. Sterilize by autoclaving), and the eggs were hatched to L1 larvae in M9 buffer without food. L1 larvae were allowed to grow to L4 larvae (36 hours) on agar plates with *E.coli* (OP50) as a food source at 20°C, and L4 larvae were then subjected to the nanoparticle dosing experiments.

### CuO Nanoparticles and sample preparation

CuO NPs (size <50 nm) were purchased from Sigma-Aldrich Chemical (St. Louis, MO, USA). The particle size of CuO nanopowder was characterized by the transmission electron microscope as <50 nm. The surface area was determined by the Brunauer–Emmett–Teller (BET) method as 29 m^2^/g. CuO NPs were dispersed in K-medium (0.032 M KCl and 0.051 M NaCl) by sonication for 90 minutes to form homogeneous suspensions. L4 nematodes were exposed for 24 hours in K-medium as controls (0 mg/mL CuO-NPs) or NP-suspensions in K-medium in the concentrations of 1, 10 or 50 µg/mL CuO-NPs, fed on OP50. Four replicates were conducted for each of the four different concentration samples. After being dosed, nematodes were harvested, rinsed with K-medium and then stored in Trizol reagent at −80 degree until RNA extraction.

### Total RNA isolation and cDNA synthesis

Total RNA was extracted from nematodes using TRI Reagent® (Ambion, Inc) according to the manufacturer's instructions with some modifications. Instead of incubating samples in TRI Reagent solution for 5 minutes at room temperature, we elongated the incubation time to 15 minutes. RNA quantification was performed with the NanoDrop ND-1000 Micro-Volume UV-Vis Spectrophotometer (NanoDrop Technologies, Wilmington, DE, USA). RNA purity was evaluated by absorbance ratios of 260/280 and 260/230. Total RNA 1 µg was used for reverse transcription with TaqMan microRNA Reverse Transcription kit (Applied Biosystems, Foster City, CA), using polyT as the reverse transcription primer.

### Quantitative PCR

Thirteen candidate reference genes were selected based on their common usage as reference genes and previous screening from *C. elegans* microarray expression data [18]. They are act-1, cdc-42, pmp-3, eif-3.C, actin, act-2, csq-1, Y45F10D.4, tba-1, mdh-1, ama-1, F35G12.2 and rbd-1. The primer information of the thirteen candidate reference genes is listed in Table 1.

**Table 1:** Primer Information of Selected Candidate Reference Genes.

Gene symbol	Locus tag	Gene description	Forward primer	Reverse primer
act-1	T04C12.6	ACTin	ACGACGAGTCCGGCCCATCC	GAAAGCTGGTGGTGACGATGGTT
cdc-42	R07G3.1	Cell Division Cycle related	AGCCATTCTGGCCGCTCTCG	GCAACCGCTTCTCGTTTGGC
pmp-3	C54G10.3	Peroxisomal Membrane Protein related	TGGCCGGATGATGGTGTCGC	ACGAACAATGCCAAAGGCCAGC
eif-3.C	T23D8.4	Eukaryotic Initiation Factor	ACACTTGACGAGCCCACCGAC	TGCCGCTCGTTCCTTCCTGG
actin	C08B11.6	Spliceosome-Associated Protein family member (sap-49)	TGGCGGATCGTCGTGCTTCC	ACGAGTCTCCTCGTTCGTCCCA
act-2	T04C12.5	ACTin	GCGCAAGTACTCCGTCTGGATCG	GGGTGTGAAAATCCGTAAGGCAGA
csq-1	F40E10.3	Calsequestrin	GCCTTGCGCTAGTGGTTGTGC	GCTCTGAGTCGTCCTCTTCCACG
Y45F10D.4	Y45F10D.4	Putative iron-sulfur cluster assembly enzyme	CGAGAACCCGCGAAATGTCGGA	CGGTTGCCAGGGAAGATGAGGC
tba-1	F26E4.8	TuBulin, Alpha family member	TCAACACTGCCATCGCCGCC	TCCAAGCGAGACCAGGCTTCAG
mdh-1	F20H11.3	Malate DeHydrogenase	TGGAGCTGCCGGAGGAATTGG	TCAGCGTTCTCAACGGCGGC
ama-1	F36A4.7	AMAnitin resistant family member	CGGATGGAGGAGCATCGCCG	CAGCGGCTGGGGAAGTTGGC
F35G12.2	F35G12.2	Hypothetical protein	ACTGCGTTCATCCGTGCCGC	TGCGGTCCTCGAGCTCCTTC
rbd-1	T23F6.4	RBD(RNA binding domain)protein	GGTCAGATTTCCGATGCGTCGCT	ACTTGCTCCAGGCTCTCGGC

Real-time quantitative PCR amplifications for reference gene candidates were carried out using 10 µL of Real-Time SYBR Green PCR master mix, 3 µL of diluted reverse transcription product, 2 µL of forward and reverse primer and 5 µL of DNase/RNase free water in a total volume of 20 µL. Amplification was carried out in a 7300 Real-Time PCR System (Applied Biosystems, Foster City, CA) with initial polymerase activation at 95°C for 10 min, followed by 40 cycles of: 95°C for 15 sec denaturation, 60°C for 60 sec for primer-specific annealing and elongation. After 40 cycles, a melting curve analysis was carried out (60°C to 95°C) to verify the specificity of amplicons.

### Data Analysis

For each primer set, standard curves made from serial dilutions of pooled cDNA were used to estimate PCR reaction efficiency (E) using the formula: E (%) = (10^[−1/slope]^−1)×100. The expression stability of the 13 candidate genes were evaluated using four commonly used algorithms, geNorm [19], NormFinder [20], BestKeeper [21], and the comparative ΔCt method [22]. The overall ranking of candidate reference genes was generated according to a method reported previously [23].

GeNorm (version 3.5) is an Excel-based applet that can be used for the analysis of gene expression stability and eventually providing two most stable reference genes. The values of transformed *Ct* (relative expression values) were transferred into the geNorm applet as input data. The expression stability value (*M* value) was calculated by the geNorm program for each candidate gene, which is described as the average pairwise variation of a single candidate reference gene to all other tested genes. A low *M* value indicates high stability in gene expression, thereby maybe ideal reference genes. Furthermore, geNorm can also provide the minimal number of reference genes required for reliable normalization. According to the pairwise variation calculation, 0.15 is commonly accepted as the cutoff, below which an additional reference gene is not required for accuracy normalization [19].

Another Excel-based software NormFinder was also used to identify reference genes for optimal normalization. This approach has the advantage of ranking the candidate reference genes both inter-group and intra-group according to their different expression stability.

Also an Excel-based software BestKeeper was used to evaluate the expression stability of candidate reference genes. This program creates an index using the geometric mean of each candidate gene's raw *Ct* values. Gene expression variation can be determined by the calculated standard deviation (*SD*) and coefficient of variance (*CV*) for all candidate reference genes based on their *Ct* values. Candidate genes with *SD* values greater than 1 were considered as inconsistent and were excluded. Then the BestKeeper program estimated the relationship between the index and the contributing reference gene by the Pearson correlation coefficient, the coefficient of determination (*r* ^2^), and the *P* value.

Moreover, the comparative *ΔCt* method was used to estimate the most stable reference genes. By comparing the relative expression of “pairs of genes” within each treatment, this method indicated the mean of standard deviation of each candidate reference genes. The candidate with lowest *SD* value was proposed to be the most stable gene and the highest *SD* value indicated the least stable gene.

## Results

L4 stage worms were exposed to four concentrations of CuO NPs (0, 1, 10, 50 µg/mL). Four biological replicates for each dose were performed. After RNA extraction and the purity analysis, the best three RNA samples from the four biological replicates in each treatment were selected to the following gene expression assay. Based on a survey of the literature, we chose 13 candidate reference genes (act-1, cdc-42, pmp-3, eif-3.C, actin, act-2, csq-1, Y45F10D.4, tba-1, mdh-1, ama-1, F35G12.2, and rbd-1) for this investigation. These included some classical “housekeeping genes” such as ama-1 (RNA polymerase II), act-1 (actin), eif-3.C (Eukaryotic Initiation Factor), tba-1 (tubulin), and some promising candidate reference genes screened from *C. elegans* microarray expression data [18]. The candidate reference genes description and their primer sets used are listed in Table 1.

### Specificity and primer efficiency in RT-qPCR reactions

The performance of each amplification primer set was tested by quantitative RT-PCR. The specificity of amplicons was confirmed by the dissociation assay following the qPCR. The presence of a single peak in the melting curve analyses for each of the 13 sets of primers indicated high specificity (data not shown).

For accurate quantification of PCR data, the amplification of all samples must have the same efficiency. The amplification efficiency (E) of the primers was determined by serial dilutions of a cDNA product solution and plotting the mean Ct values versus the logarithm transformed concentration of the dilution template. Primer efficiency is calculated according to the following formula: E (%) = (10^[−1/slope]^−1)×100. The amplification efficiencies ranged from 84.3% to 120.2% and all the correlation coefficients of R square were larger than 0.98. Thus, all primers were gene specific, and efficiencies are acceptable for further assays (Table 2).

**Table 2:** Primer amplification efficiency of the thirteen candidate reference genes.

Gene symbol	Locus tag	Amplification Efficiency (%)	R square
act-1	T04C12.6	91.0	0.9988
cdc-42	R07G3.1	100.7	0.9808
pmp-3	C54G10.3	120.2	0.9990
eif-3.C	T23D8.4	90.5	0.9999
actin	C08B11.6	84.3	0.9964
act-2	T04C12.5	87.8	0.9991
csq-1	F40E10.3	85.5	0.9997
Y45F10D.4	Y45F10D.4	88.0	0.9995
tba-1	F26E4.8	88.5	0.9995
mdh-1	F20H11.3	98.9	0.9948
ama-1	F36A4.7	91.1	0.9990
F35G12.2	F35G12.2	87.3	0.9990
rbd-1	T23F6.4	91.4	0.9994

### Expression levels of candidate reference genes

**Figure 1:**
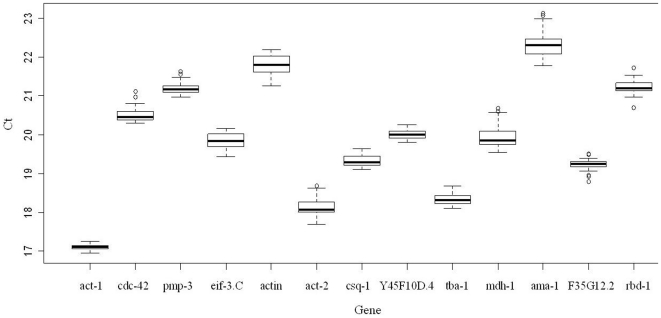
Distribution of threshold cycle (Ct) values for candidate reference genes obtained using qPCR in *C. elegans*. Boxes show the range of Ct values within each candidate gene; the black centre line indicates the median Ct; the extended upper and lower hinges indicate 75 and 25 percentiles; the whiskers show the largest/smallest Ct values that falls within a distance of 1.5 times IQR (Interquartile range) from the upper and lower hinges; outliers are shown as small circles.

The expression levels of all 13 candidate reference genes were evaluated as threshold cycle (*Ct*) values from four different concentrations of CuO NPs dosage with three biological and three technical replicates. Figure 1 shows the box plot graph of the *Ct* values of all 13 potential reference genes (for each gene n = 36). From this graph, the median *Ct* values were distributed from lowest in the case of act-1(∼17) to highest in the case of ama-1 (∼22). Also the range of the *Ct* values under different treatments showed a considerable variability among the 13 candidate reference genes. The lowest ranges of *Ct* value were act-1, Y45F10D.4, csq-1, and tba-1, indicating they are more stably expressed than others. However, a simple comparison of the raw *Ct* values is not sufficient for evaluating the expression stability of candidate reference genes. We then conducted the following four methods for verification.

### Analysis of candidate reference gene stability

To further evaluate the stability of expression of candidate reference genes, we applied four widely used algorithms to calculate the expression stability individually. An overall ranking of the expression stability was then produced. The four algorithms are geNorm [19], NormFinder [20], BestKeeper [21], and the Comparative ΔCt method [22].

#### geNorm analysis

**Figure 2:**
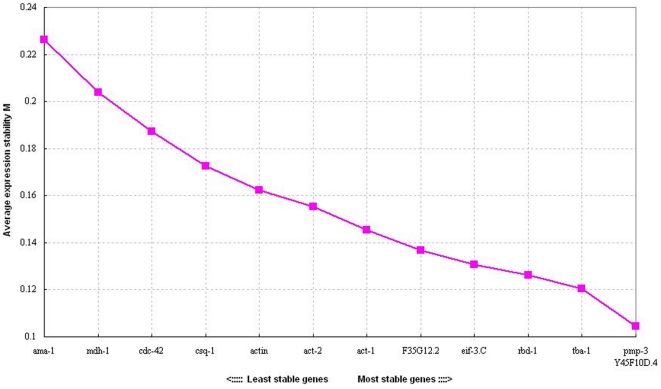
The average expression stability values of the thirteen candidate reference genes analyzed by geNorm. The lower the M value, the higher the stability.

**Figure 3:**
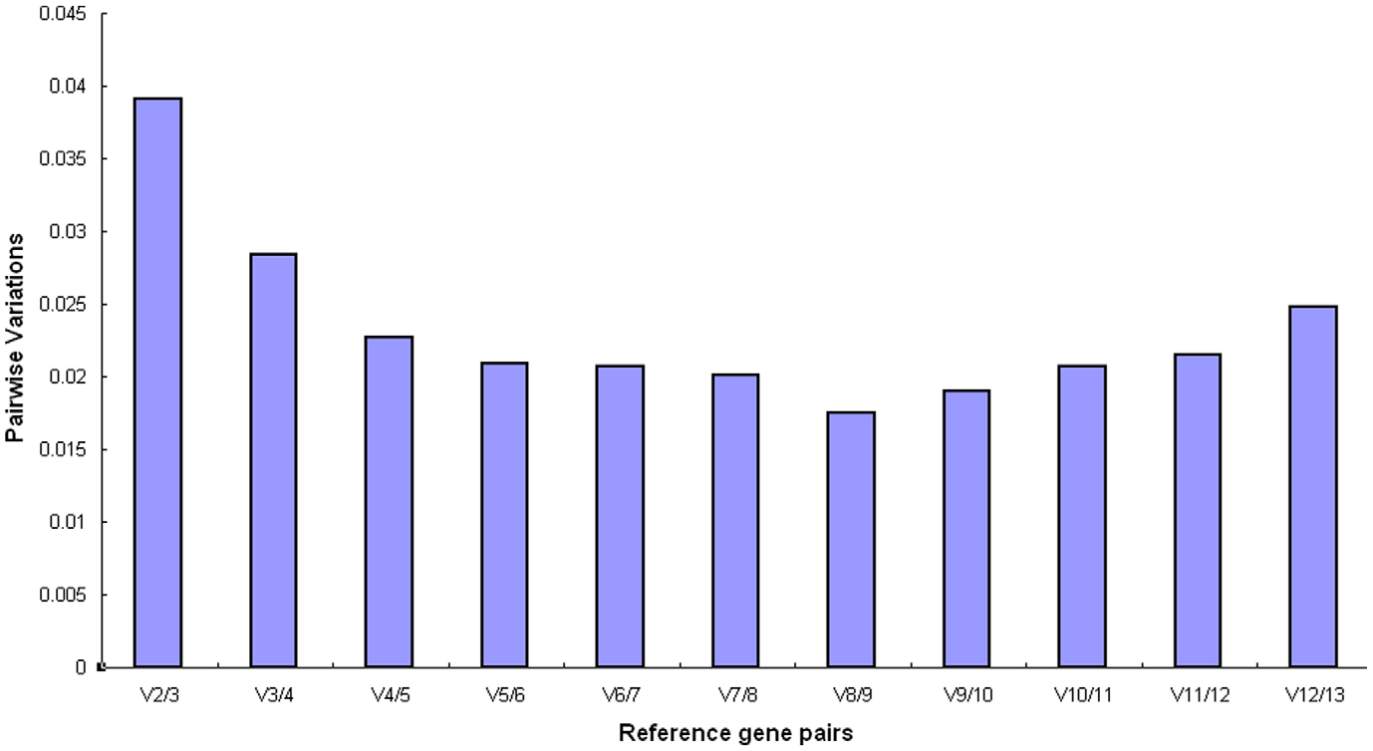
Determination of the optimal number of reference genes by geNorm analysis of the pairwise variation (V_n/n+1_).

The raw Ct values were transformed into relative quantification data. The average gene expression stability (M value) of the thirteen candidate reference genes were calculated by the geNorm applet. All candidates were ranked based on M values (Figure 2). A lower value of average expression stability M indicated more stable expression. The thirteen selected candidate genes all reached high expression stability criterion with M<1.5, the default cutoff value suggested by geNorm. In the pooled group, pmp-3 and Y45F10D.4 were the most stable genes, while ama-1 was the least stable gene under treatments of CuO NPs. From most stable (lowest M value) to least stable (highest M value), the order of the thirteen genes were pmp-3, Y45F10D.4, tba-1, rbd-1, eif-3.C, F35G12.2, act-1, act-2, actin, csq-1, cdc42, mdh-1 and ama-1 (Fig. 2 & Table 3). Moreover, we determined the optimal number of reference genes according to the pairwise variation value (V_n/n+1_ value) (Figure 3). The V_2/3_ value was smaller than the cutoff threshold of 0.15, which indicated that the top two reference genes (pmp-3 and Y45F10D.4) would be adequate in our RT-qPCR normalization during different concentrations of CuO NPs treatments, and an additional reference gene was not required.

**Table 3:** The comprehensive rankings of all thirteen candidate reference genes.

Ranking	geNorm	Normfinder	BestKeeper	Comparative ΔCt CT	Recommended comprehensive ranking
1	pmp-3 | Y45F10D.4	tba-1	act-1	tba-1	tba-1
2		pmp-3	F35G12.2	Y45F10D.4	Y45F10D.4
3	tba-1	Y45F10D.4	Y45F10D.4	pmp-3	pmp-3
4	rbd-1	rbd-1	tba-1	rbd-1	act-1
5	eif-3C	eif-3C	pmp-3	eif-3C	F35G12.2
6	F35G12.2	F35G12.2	csq-1	F35G12.2	rbd-1
7	act-1	act-1	rbd-1	act-1	eif-3C
8	act-2	act-2	cdc-42	act-2	act-2
9	actin	actin	eif-3C	actin	csq-1
10	csq-1	csq-1	act-2	csq-1	actin
11	cdc-42	cdc-42	actin	cdc-42	cdc-42
12	mdh-1	mdh-1	mdh-1	mdh-1	mdh-1
13	ama-1	ama-1	ama-1	ama-1	ama-1

#### NormFinder analysis

We also evaluated the data with NormFinder algorithm to determine the optimal reference genes for RT-qPCR normalization. This program takes intra- and inter- group variation into account for normalization factor calculations. From most stable to least stable, the rank order of the thirteen genes were tba-1, pmp-3, Y45F10D.4, rbd-1, eif-3.C, F35G12.2, act-1, act-2, actin, csq-1, cdc42, mdh-1, and ama-1 (Table 3). Compared with the result of geNorm, there were a little differences in ranking the most three stable genes. NormFinder identified tba-1 as the most stable gene, followed by pmp-3 and Y45F10D.4, and the rest of the ranking orders were the same as geNorm. These two results were relatively consistent between geNorm and NormFinder, while the differences between the two programs were expected since their statistical algorithms were distinct.

#### BestKeeper analysis

The BestKeeper applet calculates the gene expression variation for candidate genes based on each candidate gene's *Ct* values. The standard deviation (*SD*), coefficient of variance (*CV*), correlation coefficient, and the *P* value were shown in Table 4. From most stable (lowest *SD*) to least stable (highest *SD*), the rank generated by the BestKeeper were as followings: act-1, F35G12.2, Y45F10D.4, tba-1, pmp-3, csq-1, rbd-1, cdc42, eif-3.C, act-2, actin, mdh-1, and ama-1 (Table 3). Different from geNorm and NormFinder, the BestKeeper analysis highlighted act-1 and F35G12.2 as the most stable genes with the lowest *SD* (0.06 and 0.09 respectively), followed by Y45F10D.4, tba-1 and pmp-3 which were identified as the three most stable genes by geNorm and NormFinder. The ranks of mediate stable genes seemed slightly different from those ranks calculated by geNorm or NormFinder. While the least stable genes (mdh-1 and ama-1) evaluated by BestKeeper were similar as those of by geNorm and NormFinder.

**Table 4:** Expression stability evaluated by BestKeeper.

Factor	act-1	cdc-42	pmp-3	eif-3.C	actin	act-2	csq-1	Y45F10D.4	tba-1	mdh-1	ama-1	F35G12.2	rbd-1
n	36	36	36	36	36	36	36	36	36	36	36	36	36
geo Mean [Ct]	17.11	20.52	21.2	19.83	21.8	18.11	19.33	20.01	18.33	19.96	22.3	19.23	21.23
ar Mean [Ct]	17.11	20.52	21.2	19.83	21.8	18.11	19.33	20.01	18.33	19.96	22.3	19.23	21.23
min [Ct]	16.95	20.29	20.97	19.45	21.26	17.7	19.1	19.8	18.1	19.54	21.77	18.79	20.7
max [Ct]	17.27	21.12	21.63	20.17	22.2	18.69	19.64	20.25	18.68	20.68	23.13	19.51	21.71
std dev [+/− Ct]	0.06	0.14	0.12	0.17	0.21	0.19	0.13	0.1	0.12	0.23	0.25	0.09	0.14
CV [% Ct]	0.36	0.7	0.59	0.87	0.97	1.04	0.66	0.48	0.67	1.15	1.11	0.49	0.66
min [x-fold]	−1.12	−1.17	−1.17	−1.31	−1.45	−1.33	−1.17	−1.15	−1.17	−1.33	−1.44	−1.36	−1.45
max [x-fold]	1.11	1.52	1.34	1.26	1.32	1.49	1.24	1.19	1.27	1.65	1.78	1.21	1.4
std dev [+/− x-fold]	1.04	1.11	1.09	1.13	1.16	1.14	1.09	1.07	1.09	1.17	1.19	1.07	1.1
coeff. of corr. [r]	0.198	0.231	0.883	0.866	0.843	0.878	0.001	0.741	0.888	0.696	0.418	0.506	0.857
p-value	0.247	0.174	0.001	0.001	0.001	0.001	0.94	0.001	0.001	0.001	0.011	0.002	0.001

#### Comparative ΔCt method analysis

Furthermore, we used the comparative ΔCt method to estimate the most stable reference genes. The result was similar to that of NormFinder and geNorm. The only differences were the ranking order of the first three reference genes. From most to least stable genes, the rankings were tba-1, Y45F10D.4, pmp-3, rbd-1, eif-3.C, F35G12.2, act-1, act-2, actin, csq-1, cdc42, mdh-1, and ama-1 (Table 3).

#### Final ranking of candidate reference genes

Given the specific features of each algorithm, all four sets of results should be taken into consideration to produce the final ranking. A method previously described by Chen *et al*. [23] was used to give an overall ranking of candidate reference genes. Briefly, the geometric means of the four ranking numbers of each gene were calculated, and then candidate reference genes were ranked according to the geometric mean, the gene with smaller geometric mean being the most stable reference gene. The recommended comprehensive rankings were given in Table 3. As a result, tba-1, Y45F10D.4, and pmp-3 turned out to be the most stable genes in different treatment groups. Therefore these three genes are recommended to be used as reference genes for RT-qPCR normalization under the treatment of CuO NPs and potentially other metal oxide NPs in *C. elegans*.

## Discussion

RT-qPCR has been a powerful tool for quantification of mRNA transcripts, especially for the detection of weakly expressed transcripts due to its high sensitivity. Normalization with stable reference genes is essential for accurate interpretation of variations in the RT-qPCR data. A reference gene should be expressed at a stable level regardless of the experimental treatments. Using non-validated reference genes may lead to inaccurate conclusions. In this study, we have evaluated thirteen candidate reference genes and validated a set of reference genes which are suitable for RT-qPCR gene expression analysis under the CuO NPs exposure in *C. elegans*.

When analyzing the stability of all the 13 candidate reference genes, we applied four commonly used programs: geNorm, NormFinder, BestKeeper, and the comparative ΔCt method. After analysis of the RT-qPCR data, geNorm identified that pmp-3 and Y45F10D.4 were the two most stable reference genes, followed by tba-1. Both NormFinder and the Comparative ΔCt method determined that tba-1 was the most stable reference gene, followed by two reference genes pmp-3 and Y45F10D.4. By contrast, BestKeeper highlighted act-1 and F35G12.2 as the first two most stable reference genes, followed by three genes Y45F10D.4, tba-1, and pmp-3. From 4^th^ to 13^th^ place, geNorm, NormFinder, and the Comparative ΔCt method gave the same results of ranking. Mdh-1 and ama-1 were the least stable candidate reference genes assessed by all four programs, therefore are not recommended for use in metal oxide NPs gene expression studies. The results from geNorm, NormFinder, and the comparative ΔCt method assessment were more consistent with each other than with the BestKeeper method. The overall ranking of the 13 candidate reference genes was determined by the comprehensive results from all four algorithms. Finally, we recommended that tba-1, Y45F10D.4, and pmp-3 are the most stable ones which could be used for RT-qPCR assay of metal oxide effects on *C. elegans*. Although any single gene of these three selected genes may be sufficient and could serve the normalization purpose, combination of two or three reference genes would give better and more accurate biological conclusion in gene expression analysis by RT-qPCR.

Our results indicated that some of the commonly used reference genes such as act-1, act-2, or ama-1 may not be the optimal choice for testing NPs effects on *C. elegans*. A previous report regarding reference genes selection in *C. elegans* also showed that pmp-3 and Y45F10D.4 were among the most stably expressed genes, regardless the different development stages or different strains of *C. elegans*. Also, cdc-42 was identified as one of the most stable reference gene [18]. However, cdc-42 was one of most variable reference genes in our study. In conclusion, this present study demonstrated again the importance of reference gene selection for RT-qPCR analysis of new materials. We identified tba-1, Y45F10D.4, and pmp-3 as the most reliable reference genes, which would be useful in future toxicological studies of nanoparticles.
